# Odorant Receptor OR2C1 Is an Essential Modulator of Boar Sperm Capacitation by Binding with Heparin

**DOI:** 10.3390/ijms24021664

**Published:** 2023-01-14

**Authors:** Xiang Yuan, Yihan Wang, Malik Ahsan Ali, Ziyue Qin, Zhihua Guo, Yan Zhang, Ming Zhang, Guangbin Zhou, Jiandong Yang, Lei Chen, Linyuan Shen, Li Zhu, Changjun Zeng

**Affiliations:** 1 Key Laboratory of Livestock and Poultry Multi-Omics, Ministry of Agriculture and Rural Affairs, College of Animal Science and Technology, Sichuan Agricultural University, Chengdu 611130, China; 2 Farm Animal Genetic Resources Exploration and Innovation Key Laboratory of Sichuan Province, College of Animal Science and Technology, Sichuan Agricultural University, Chengdu 611130, China; 3 Department of Theriogenology, Faculty of Veterinary Science, University of Agriculture, Faisalabad 38000, Punjab, Pakistan

**Keywords:** olfactory receptor, *OR2C1*, heparin, boar sperm, capacitation

## Abstract

Heparin, a class of glycosaminoglycans (GAGs), is widely used to induce sperm capacitation and fertilization. How heparin induces sperm capacitation remains unclear. Olfactory receptors (ORs) which are G protein-coupled receptors, have been proposed to be involved in sperm capacitation. However, the interaction between ORs and odor molecules and the molecular mechanism of ORs mediating sperm capacitation are still unclear. The present study aimed to explore the underlying interaction and mechanism between heparin and ORs in carrying out the boar sperm capacitation. The results showed that olfactory receptor 2C1 (OR2C1) is a compulsory unit which regulates the sperm capacitation by recognizing and binding with heparin, as determined by Dual-Glo Luciferase Assay and molecular docking. In addition, molecular dynamics (MD) simulation indicated that OR2C1 binds with heparin via a hydrophobic cavity comprises of Arg3, Ala6, Thr7, Asn171, Arg172, Arg173, and Pro287. Furthermore, we demonstrated that knocking down *OR2C1* significantly inhibits sperm capacitation. In conclusion, we highlighted a novel olfactory receptor, OR2C1, in boar sperm and disclosed the potential binding of heparin to Pro287, a conserved residue in the transmembrane helices region 7 (TMH7). Our findings will benefit the further understanding of ORs involved in sperm capacitation and fertilization.

## 1. Introduction

Mammalian olfactory receptors (ORs) are classified as G protein-coupled receptors (GPCRs), which are primarily located in olfaction mediating sensory cells [1,2,3]. ORs initiate a series of biochemical signaling transduction by specifically recognizing odor molecules that convert the chemical signals of odor molecules into electrical signals to identify various odors [4]. However, ORs are also widely distributed in non-olfactory tissues and cells, including heart [5], kidney [6], liver [7], testis [8], oocytes [9], and mammalian sperm [10,11,12]. In human sperm, OR6B2 and OR2W3 are located in the acrosome and flagellum of sperm respectively [13]. Moreover, OR51E2, OR4S1, OR4C13, and OR1I1 are located in the acrosome, mid-piece, and flagellum of sperm [12,14]. The ORs located in the flagellum and mid-piece of sperm are most likely involved in sperm chemotaxis, while those distributed in sperm acrosome are more likely to participate in sperm acrosome reaction and capacitation, suggesting different functions in response to different cellular patterns of ORs distribution.

Sperm capacitation is an indispensable event before fertilization, characterized by a set of physiological and chemical changes that enables sperm to penetrate zona pellucida of oocytes [15,16]. Many studies suggest that litter size and fertility showed significant correlations with the proportion of capacitated spermatozoa, and defects in sperm capacitation can also reduce fertility [17,18,19]. For instance, defects in cystic fibrosis transmembrane conductance regulator (CFTR), which regulates bicarbonate production is crucial for sperm capacitation, have also been identified to seriously undermine male fertility [20]. To further improve the fertility of males, proper understanding of sperm capacitation mechanism is required, because only capacitated spermatozoa are capable of fertilizing oocytes both in vitro and in vivo. It is generally believed that dramatic changes occur during sperm capacitation and are manifested as the deconstruction of sperm membrane [21], increase in intracellular pH (pH_i_ ) [22], influx of Ca^2+^ [23], membrane hyperpolarization [24], and tyrosine phosphorylation [25]. Capacitation is induced by different factors in in-vitro media or female reproduction tract, such as heparin [26], bicarbonate [27], bovine serum albumin (BSA) [28], and calcium ions [29]. However, the molecular mechanism about how different substrates cause sperm’s capacitation has not been fully unveiled. CatSper channel and bicarbonate transporters are marked as the main gateways to allow the influx of calcium (Ca^2+^) and bicarbonate ions (HCO_3_^−^), which are responsible for activating the cAMP/cGMP signaling pathway by activating soluble adenylate cyclase (sAC) [30]. In addition to Ca^2+^ and HCO_3_^−^, some macromolecules, such as heparin (glycosaminoglycan), can only initiate the cAMP/PKA signaling pathway by mediating the efflux of cholesterol [31], which is similar to the action of BSA [32]. The difference is that heparin binds and removes the heparin-binding proteins that adhere to the surface of sperm from seminal plasma, disrupting lipid rafts and capacitation indirectly [33]. However, it is unclear whether some proteins interact with heparin on the sperm membrane to initiate the regulation of capacitation or not. Our previous report discovered that the differentially expressed miRNAs and mRNAs were highly enriched in the olfactory transduction pathway when boar sperm was treated with heparin [34]. In addition, sperm membrane signal transduction based on cAMP and Ca^2+^ may participate in quality regulation of frozen-thawed boar and giant panda sperm, which suggests that olfactory receptors may be involved in the regulation of sperm capacitation or cryo-capacitation-like changes [35].

Heparin belongs to glycosaminoglycans (GAGs), which was identified as a new class of odorants in fish that activate the olfactory bulb and trigger fear responses via a specialized circuit [36]. It was reported that the expressions of GAGs play a key role in the functioning of mouse olfactory epithelium (OE) [37], which transmits the signal of odorants. The alteration in the expression of GAGs contributes to olfactory receptor neuron (ORN) dysregulation and abnormal olfactory molecules identification in the pathophysiology of schizophrenia [38]. GAGs can also promote the maturation of human ORN [39] and activate signaling pathways, which are essential for the proliferation and self-renewal of neural stem/progenitor cells [40]. Intriguingly, heparin-like GAG is secreted by the female reproductive tract and mediates sperm function and interaction between sperm and oocytes [41], such as capacitation and fertilization, respectively. Defects in sperm capacitation lead to poor fertility and even infertility. The administration of low doses of heparin may increase clinical pregnancy and live birth rates in women undergoing in vitro fertilization (IVF) or intracytoplasmic sperm injection (ICSI) [42]. The deconcentrated sperm produced by heparin-glutathione pretreatment also increased the efficiency of ICSI in cattle [43]. Consequently, it is vital to elucidate the mechanism of sperm capacitation induced by heparin for improving fertility and treating infertility. In this study, we comprehensively demonstrated that heparin-like GAG induces sperm capacitation by binding to a specific olfactory receptor, OR2C1, in the plasma membrane via Dual-Glo Luciferase Assay, molecular docking and molecular dynamics (MD) simulation. Our findings will benefit the further understanding of the interaction and molecular mechanism of ORs and capacitation-inducing factors.

## 2. Results

### 2.1. Effects of Different Substrates on Sperm Capacitation and mRNA Expression of Olfactory Receptor

**Figure 1:**
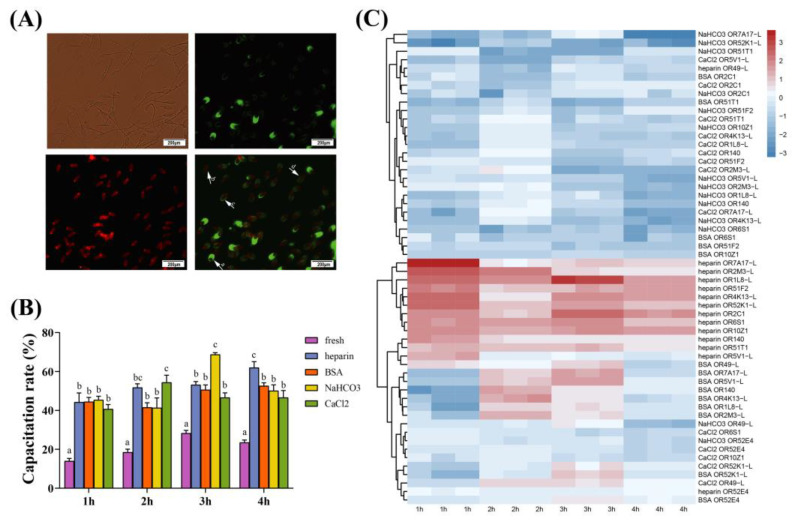
Sperm capacitation and mRNA expression profiling of ORs with different substances. (**A**) Assessment of the acrosomal status by a pisum sativum agglutinin (PSA)/propidium iodide (PI) staining assay. a: integral acrosome (green, PI−/PSA+), b: dead sperm (red, PI+/PSA−), c: acrosomal reaction (green/red, PI+/PSA+), d: damaged acrosome and incomplete membrane (green/red, PI+/PSA+). The capacitation rate is equal to the sum of the C group divided by the total sperm. (**B**) Capacitation rate of sperm. (**C**) Hierarchical clustering diagram of relative mRNA expression levels of ORs in boar sperm treated with NaHCO_3_, BSA, CaCl_2_, and heparin for 1 h–4 h. The *X*-axis represents the processing time, and the *Y*-axis represents the log10 (relative expression). The data are representative of at least three independent experiments (mean ± SEM).

The sperm capacitation rate and mRNA expression of the olfactory receptors were evaluated from 1 h to 4 h. The capacitation rates in the heparin, BSA, NaHCO_3_, and CaCl_2_ were all significantly higher at every time point (*p* < 0.01) than the fresh group (Figure 1B). Especially at 4 h, the capacitation rates in heparin were significantly higher (*p* < 0.01) than other treatments.

In addition, the hierarchical clustering diagram disclosed that the mRNA expression patterns of olfactory receptor genes in sperm treated with heparin were considerably different from those in other treatments (Figure 1C). Heparin broadly upregulated the relative expression of *OR2C1, OR10Z1, OR6S1, OR4K13-L, OR52K1-L, OR51F2, OR1L8-L, OR7A17-L, OR51T1, OR140*, and *OR2M3-L* from 1 h to 4 h; in contrast, BSA, CaCl_2_ and NaHCO_3_ generally downregulated the relative expression of olfactory receptors. Furthermore, BSA rarely upregulated the relative expression of olfactory receptors in 2 h and 3 h. In the heparin group, the relative expression of most olfactory receptors in 1 h has a higher elevation compared to 2 h, 3 h, and 4 h. The down-regulated heparin group is only found in *OR49-L* and *OR52E4*. Moreover, extensive down-regulation can be found in the BSA, CaCl_2_ and NaHCO_3_ groups.

### 2.2. Homology Modelling and Molecular Docking of OR2C1 and Heparin

**Figure 2:**
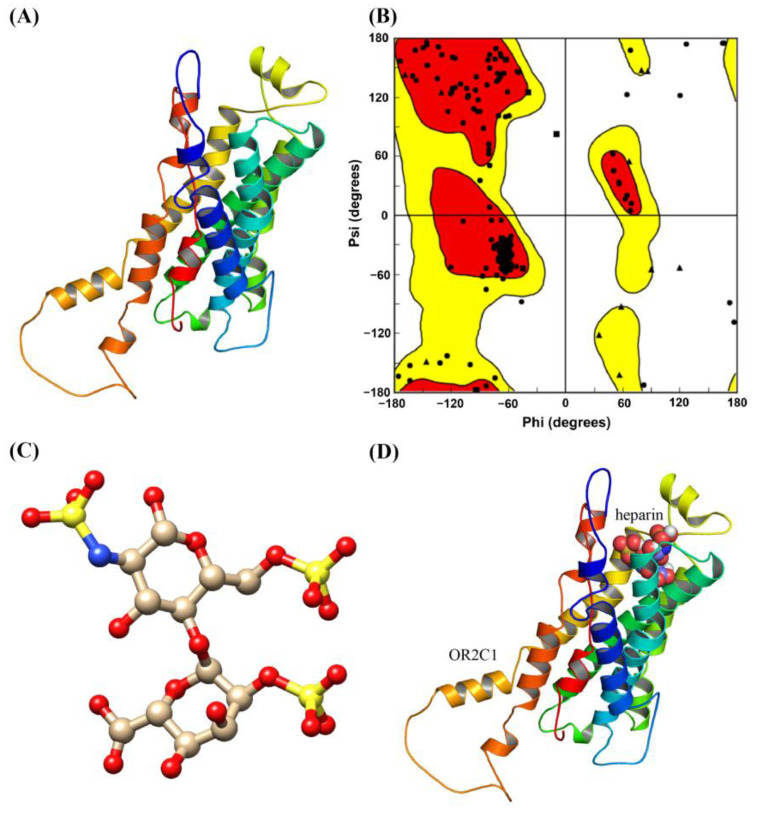
Homology modelling and molecular docking of OR2C1 and heparin. (**A**) Homology model of OR2C1 protein; (**B**) Ramachandran plot of OR2C1 protein (the Ramachandran plot is mainly divided into three areas: allowed area (red area), maximum allowed area (yellow area), and not allowed area (blank area)); (**C**) Structure of heparin; (**D**) The predicted binding model of OR2C1 and heparin.

The models of the OR2C1 receptors were constructed while obeying the energy rules of stereochemicals (Figure 2A), which have similar structures to other reported ORs [44,45,46]. Of the amino acid residue dihedral angles in the models of the OR2C1 receptors, 98.2% are endowed with a reasonable range. Among them, 96.4% of the amino acids were located in the permissive region, and only 1.8% of the amino acids were located in the forbidden region of the torsion angle (Figure 2B). After simulating the structure of heparin and olfactory receptors through homology modeling, we performed molecular docking prediction of binding between heparin and ORs (Table S1). The result showed that heparin primarily bound to a cavity formed by transmembrane helices at the N terminus of the OR2C1 receptor (Figure 2D). Furthermore, the lowest predicted binding energy required by heparin was found in the OR2C1 receptor compared to other receptors (−6.79 kcal/mol); in consequence, OR2C1 was the top-ranked binder for heparin (Table S2).

### 2.3. Molecular Dynamics Simulation for OR2C1 and Heparin

**Figure 3:**
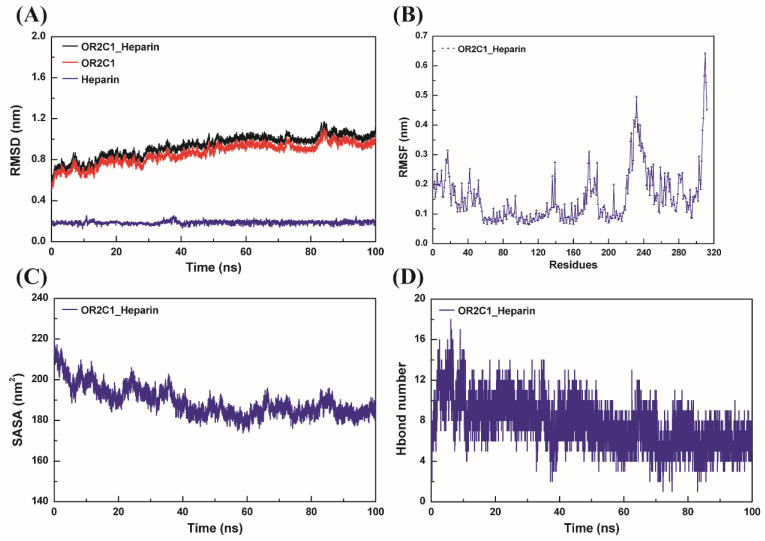
Molecular dynamic simulation between OR2C1 and heparin for 100 ns in central cavity. (**A**) RMSD of the residues of modelled OR2C1 structure and heparin; (**B**) RMSF of the residues in the system of OR2C1 binding heparin; (**C**) SASA; (**D**) number of H-bonding contacts with ligand.

To further investigate the possible binding of OR2C1 to heparin, molecular dynamics simulations were performed. As shown in Figure 3A, the rise in the root mean square deviation (RMSD) in protein/ligand OR2C1_Heparin complex only occurred in the early stage of simulation and was stable from about 40 ns with the average value of 1.008 ± 0.044 nm. On the other hand, we analyzed the root mean square fluctuation (RMSF) value of all amino acids in the whole system in Figure 3B. In the OR2C1_Heparin system, the RMSF of the C-terminal amino acid of the OR2C1 protein was significantly higher than that in other regions. In addition, RMSF values also fluctuated greatly in the region of V225-C241 located in a loop at the bottom of the OR2C1 protein. Nevertheless, the RMSF of the whole OR2C1 protein did not fluctuate significantly due to the binding of heparin. The solvent accessible surface area (SASA) of OR2C1 protein was measured in Figure 3C, which has a larger value at the beginning of the simulation which swiftly decreases before 40 ns, and then tends to be stable at about 40 ns. Furthermore, the SASA of the stabilized structure was smaller than that of the initial structure, and the structure of the complex was more compact.

The results showed that the total free binding energy between OR2C1 protein and heparin, measured at a stable RMSD interval of 80–100 ns, was negative (ΔG_bind_ = −40.744 kcal/mol) (Table 1). Among these, the electrostatic interaction of the system, including electrostatic interaction under vacuum (ΔG_ele_ = −318.485 kcal/mol) and polar solvation energy (ΔG_PB_ = 318.227 kcal/mol), have poor binding energy (ΔG_vdw_ + ΔG_np_ = −0.258 kcal/mol). Higher binding energy was observed in non-polar interactions including van der Waals force interaction (ΔG_vdw_ = −35.293 kcal/mol) and non-polar solvation free energy (ΔG_np_ = −5.193 kcal/mol). Hydrogen bonds between the OR2C1 protein and heparin over the simulation time have been counted and are shown in Figure 3D. The number of hydrogen bonds between OR2C1 protein and heparin decreased slightly when the whole complex structure became stable. At this time, the average number of hydrogen bonds is 6.3, which is significantly less than 10.6 during 0–10 ns.

**Figure 4:**
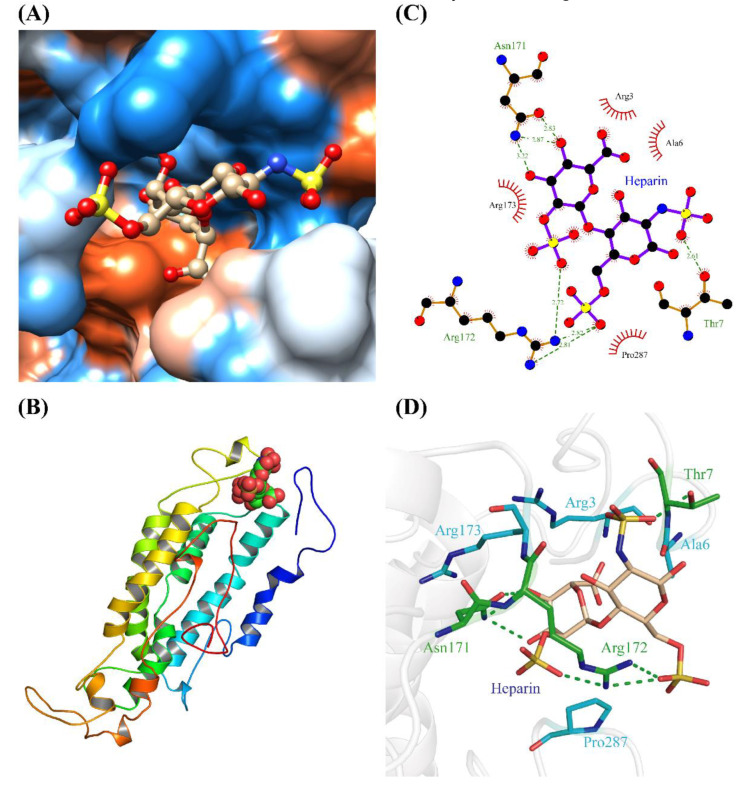
Interaction maps of binding pattern between heparin and OR2C1. (**A**) Heparin binding to the hydrophilic (blue) and hydrophobic (orange) surface of OR2C1; (**B**) position of heparin in 3D structure of OR2C1 protein; (**C**) ligand–protein interactions between heparin and OR2C1 with the residues that form H-bonding interaction (green) and hydrophobic interaction (red gear); (**D**) 3D binding pattern diagram of heparin and OR2C1, the H-binding are shown in green dotted line.

To further confirm the main interaction region and key amino acids between OR2C1 protein and heparin, the complex conformation after MD simulation was extracted. As shown in Figure 4, a hydrophobic cavity consisting of Arg3, Ala6, Thr7, Asn171, Arg172, Arg173, and Pro287 is the place where heparin binds to OR2C1 protein. The phosphate group on the heparin molecule bind with Thr7, Asn171, and Arg172 in OR2C1 to form hydrogen. Furthermore, a strong hydrophobic effect also formed in heparin with surrounding hydrophobic amino acids (Arg3, Ala6, Arg173, and Pro287). Among this, Pro287 exist in the transmembrane helices region 7 (TMH7) of OR2C1 (Figures S1 and S2). All of these intermolecular forces further enhanced the affinity between heparin and the OR2C1 receptor.

### 2.4. Activation of OR2C1 with Heparin

**Figure 5:**
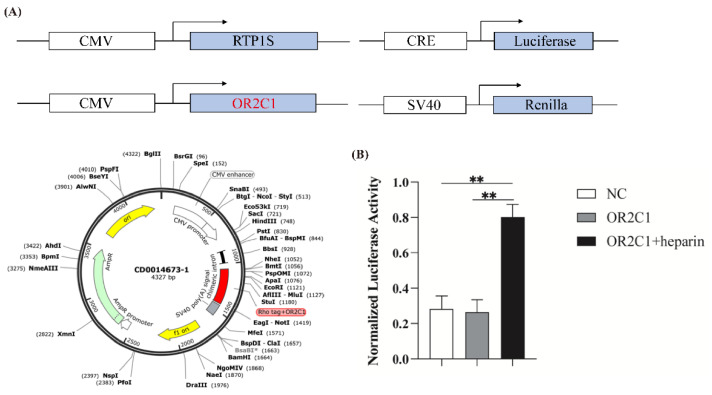
Construction of plasmid and Dual-Glo Luciferase Assay. (**A**) The structure of recombinant plasmid, including OR2C1, RTPIS, Luciferase, and Renilla; The genes of *RTP1S* and *OR2C1* are driven by cytomegalovirus promoter (CMV), Luciferase (Luc) is driven by the cAMP response element promoter (CRE), and Renilla (RL) is driven by the constitutive promoter (SV40). (**B**) Dual-Glo Luciferase Assay. The formula ([(Luc/RL)_N_ − (Luc/RL)_lowest_]/[(Luc/RL)_highest_ − (Luc/RL)_lowest_]) was used to normalize the luciferase activity. ** *p* < 0.01. The data are representative of at least three independent experiments (mean ± SEM).

The heterologous expression of ORs in Hana3A cells is generally used to verify the binding of ectopic ORs and ligands [45]. The structure of recombinant plasmid was shown in Figure 5A. The results showed that the normalized luciferase activity in transfected *OR2C1* with heparin cells was significantly higher than that in the transfected *OR2C1* group and the NC group (*p* < 0.01) (Figure 5B). In addition, no significant difference (*p >* 0.05) was observed between the transfected *OR2C1* group and the NC group. Therefore, these results further demonstrated that heparin can activate *OR2C1*.

### 2.5. Regulation of Heparin-Activated OR2C1 in Sperm Capacitation

**Figure 6:**
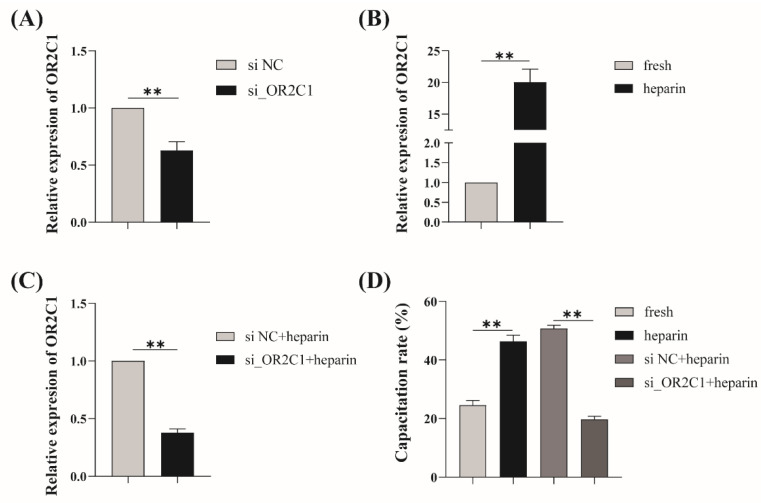
Histograms of relative mRNA expression of *OR2C1* and sperm capacitation rate with si-*OR2C1*. (**A**) The relative mRNA expression of *OR2C1* in si_*OR2C1* and (**B**) treated with heparin group; (**D**) the sperm capacitation rate of si_*OR2C1* and further treated with heparin group. The *Y*-axis displays the relative expression of the *OR2C1* gene (**A**–**C**) and sperm capacitation rate (%, **D**), respectively. Non-targeting siRNA oligonucleotides were transfected as negative control (si NC). ** *p* < 0.01. The data are representative of at least three independent experiments (mean ± SEM).

To verify the role of OR2C1 in heparin-induced capacitation in boar sperm, knocking down *OR2C1* (si_*OR2C1*) significantly (*p* < 0.01) decreased the mRNA expression of *OR2C1* (Figure 6A). After the addition of heparin, the relative expression of *OR2C1* was significantly increased (*p* < 0.01) compared with the fresh group (Figure 6B). The mRNA expression of *OR2C1* in knocking down *OR2C1* with the heparin group was significantly decreased (*p* < 0.01) compared with that in the non-targeting siRNA (si NC) with heparin group (Figure 6C).

In addition, a significantly increased (*p* < 0.05) capacitation rate was found in the heparin group compared with the fresh group (Figure 6D). Meanwhile, the si_*OR2C1* with heparin group was significantly decreased (*p* < 0.01) in comparison to the si NC with heparin group.

## 3. Discussion

Heparin is a common glycosaminoglycan (GAG) type of polysaccharide in ovarian follicular fluid and is widely used to induce sperm capacitation [47]. In addition, BSA, NaHCO_3_, and CaCl_2_ are also reported as capacitation-inducing factors, and are widely used to induce sperm capacitation. In the present study, surprisingly, heparin appears to have a stronger facilitated effect on boar sperm capacitation. Particularly at 4 h, the sperm capacitation rate induced by heparin was markedly higher than that of other capacitation factors. These results indicate that the pathway and mode of action of these different capacitation inducing factors may be different. For instance, the uptake of calcium and bicarbonate through the specific transporters flow into the sperm membrane stimulates sAC and cAMP elevation and further initiates sperm capacitation, respectively [48,49,50]. In contrast, both macromolecular BSA and heparin cannot pass through the sperm membrane; they reposition lipid rafts by enhancing membrane fluidity to induce capacitation [50,51,52]. This may explain why the relative expression of olfactory receptor genes, which code for membrane receptor proteins, were up-regulated when treated with heparin and BSA, but not when treated with calcium and bicarbonate. It is worth noting that the effects of heparin on olfactory receptor gene expression were substantially broader than that of BSA due to their different interaction mechanisms. It was reported that BSA induces cholesterol efflux by binding directly to cholesterol in sperm membranes, while heparin removes seminal plasma adhesive proteins from the sperm membrane surface which prevent premature capacitation, and indirectly increases plasma membrane fluidity [31]. Detailed studies demonstrated that a group of heparin-binding proteins bind to the sperm and are present in the seminal plasma at ejaculation, which restricts the sperm’s fertilizing capacity. Heparin binds to this kind of protein and removes them from sperm to induce sperm capacitation, such as PDC-109 in bull [53] and wheat germ agglutinin-reactive protein in boar [33]. However, the regulatory mechanism activated by heparin after this process is not fully understood.

ORs were generally reported to exist in sperm [54] and widely believed to be involved in sperm chemotaxis, further regulating sperm fertilization and capacitation [10,11,12,14,55]. Odorants can be unique ligands that interact with ORs to reinforce these processes. Spehr found a special odorant bourgeonal that can bind with hOR17-4 to trigger transient Ca^2+^ response and modify sperm kinetic models [55,56]. Human sperm exhibited elevated swim speeds and directed movements after reacting to bourgeonal, exposing that bourgeonal is not only a compelling chemoattractant but also an effective swimming stimulant for sperm that navigate them towards oocytes. In addition, Lyral as a cognate ligand of olfactory receptor MOR23 can induce the influx of cellular Ca^2+^ by activating MOR23, leading to the modulation of motility and chemotaxis in mouse sperm [55]. Except these exogenous odorants, the endogenous ligands 5A-androst-16-en-3-one and 4-hydroxy-2,5-dimethyl-3(2H)-furanone found in follicular fluid activate chemotaxis of sperm in humans toward the oocytes and act on ORs such as OR4D1 and OR7A5 on their membranes [57]. So far, numerous studies have verified that odorant-induced Ca^2+^ signals are indeed present in sperm [58]. Multiple tested OR ligands have been monitored using single-cell calcium imaging experiments, and the results indicated that most of them could transiently induce Ca^2+^ influx [12]. The influx of Ca^2+^ is a prerequisite for sperm capacitation which proves ORs function as an upstream switch in sperm capacitation according to their mode of interaction [59]. Our previous study showed that the differentially expressed genes were mainly enriched in the olfactory transduction signaling pathway in fresh and heparin-induced capacitated boar sperm, implying the potential regulation of ORs in this way [34]. As a consequence, in this study, we further aimed to elucidate the underlying molecular mechanism of ORs in heparin-induced sperm capacitation.

It is generally accepted that ORs could be activated by traditional odorants; however, the ligands of ORs are not only limited to them. For example, as a male pheromone in sea lamprey, spermine can act as a novel ligand on OR TAAR384 to attract ovulatory females [60]. In this study, our results showed that heparin significantly up-regulates olfactory receptor gene expression, which implies the potential role of heparin in inducing sperm capacitation. In order to further explore whether heparin directly binds ORs as a novel odorant or not, we performed subsequent experiments and obtained a positive result that heparin is able to join with various ORs. Different ORs have remarkable affinities for each substance; for example, MOR107-1 is activated only by the (-) enantiomer of fenchone and MOR271-1 is not [61]. Our results have verified that OR2C1 displays the highest binding affinity for heparin (Figure 2). In mammals, ORs are also highly conserved proteins that generally bind to ligands in the cavity formed by the transmembrane helix at their N-terminal [44,45,62]. Similarly, molecular docking and MD simulations uncovered that heparin binds to the N-terminal cavity in OR2C1 predominantly through an alternating interplay of hydrogen bonding and a strong hydrophobic effect among certain residues. Non-polar interactions (ΔG_vdw_ + ΔG_np_ = −40.486 kcal/mol) were inferred to be the main driving force between OR2C1 protein and heparin. Moreover, ORs also contain conserved residues which are crucial for OR activation and specificity control of olfactory G protein [45,63]. The highly conserved proline residues always exist in the TMH7 of ORs such as Pro285 and Pro281 in OR1A1 and Olfr43 which cause a pronounced kink in TMH7 [46]. We similarly detected Pro283 and Pro287 in the TMH7 region of the OR2C1 protein, which were analogous to conserved residues. In particular, Pro287 formed a strong hydrophobic interaction with heparin, which increased the affinity of OR2C1 and heparin.

OR2C1 was a putative odorant receptor of sperm cells in many species, such as boar, bovine, horse, and goat, which was confirmed from UniProt [64]. Limited reports have found that OR2C1 favor the survival of isocitrate dehydrogenase wild-type glioma, certifying that OR2C1 was one of the therapeutic targets for glioma [65]. A novel link between heparin and OR2C1 in sperm emerges from this study. Heparin-like GAGs influence sperm-oocyte interaction by interacting with oviduct-specific glycoprotein in the development of zona pellucida modifications [66]. It has been reported that GAGs are widely expressed and modulate olfactory neuronal homeostasis in mouse and human ORN, suggesting that GAGs can bind with ORs like an activator [37,38,39]. Whether olfactory receptors mediate the capacitation effected by heparin-like GAGs during sperm in the oviduct remains poorly understood in part because of the difficulty of experiments in the female reproductive tract. However, our results showed that after the addition of exogenous siRNA, the relative expression level of *OR2C1* mRNA was significantly reduced compared with that in the NC group, demonstrating its ability to target degradation of *OR2C1* mRNA already present in mature sperms (Figure 6A). At the same time, although mature sperm are generally considered to be transcriptionally silent, previous studies have found that heparin can rapidly transform mature sperm into a capacitated state, and there is a huge transcriptomic difference before and after the process [34], which is manifested as the up-regulation of olfactory receptor-related genes. The phenomenon of breaking the gene silencing state in mature sperm by heparin also seemed to be demonstrated by our results that the sperm capacitation rate and the relative expression of *OR2C1* mRNA in the heparin group was significantly increased (Figure 6B, D). Based on this, after the introduction of exogenous siRNA, the prominent decrease in relative expression of *OR2C1* mRNA, accompanied by a significant decrease in the sperm capacitation rate has been observed (Figure 6C, D). These results have confirmed the potential role of OR2C1 in sperm capacitation induced by heparin in vitro, which inspires us to carry out deeper research.

## 4. Materials and Methods

### 4.1. Animal Ethics Statement

All animal procedures involving animal treatments were performed in accordance with the Institutional Animal Care and Use Committee in the College of Animal Science and Technology, Sichuan Agricultural University, Sichuan, China (under permit no. 2020202040), which conforms to the Regulations of the Administration of Affairs Concerning Experimental Animals (Ministry of Science and Technology, China, 2017).

### 4.2. Materials

Trizol LS Reagent kit (Invitrogen, Carlsbad, CA, USA), TaKaRa PrimeScript RT Reagent Kit (Takara Biotech, Beijing, China), Fluorescein isothiocyanate isomer conjugated with PSA (FITC-PSA) ( Sigma, Saint Louis, MO, USA), PI (Sigma, Saint Louis, MO, USA), Roswell Park Memorial Institute Medium (RPMI Medium) (Gibco, Grand Island, NY, USA), Fetal bovine serum (FBS) (Pan, Aidenbach, Germany), TransIntro^TM^ PL Transfection Reagent (Tran, Beijing, China), CD293 (Invitrogen, Carlsbad, CA, USA), Dual-Glo Luciferase Assays System (Promega, Madison, WI, USA), non-targeting siRNA oligonucleotides (siRNA NC) (Ribobio, Guangzhou, China), silencing siRNA targeted against the OR2C1 gene (siRNA OR2C1) (Ribobio, Guangzhou, China).Unless stated, all chemicals used were purchased from Sigma (Sigma, Saint Louis, MO, USA). BTS (37 g glucose, 3 g trisodium citrate, 1.25 g Na_2_-EDTA, 1.25 g NaHCO_3_, 0.75 g KCl, 0.6 g/L penicillin G sodium, and 1.0 g/L dihydrostreptomycin; diluted to 1 L).

### 4.3. Sperm Collection and Treatment

Ejaculates (n = 10) were collected from healthy and sexually active Yorkshire boars, provided by Pengzhou Jinzhu farm (Pengzhou, Sichuan, China). Fresh ejaculates were transported in a portable refrigerator (17 °C) within 1 h to the lab. All the samples were tested through standard assessment of semen quality and only those with normal morphology, more than 90% motility, 80% viability, and higher than 1 × 10^8^ mL^−1^ concentration in subsequent experiments were used.

All semen (n = 10) were mixed to a pool, and then equally divided into four aliquots. Firstly, one aliquot was centrifugated (1500 rpm/min, 5min) and immediately suspended in BTS at 37 °C for 1–4 h, which was regarded as fresh group. A second aliquot was incubated in BTS with heparin, BSA, NaHCO_3_, CaCl_2_ for capacitation, respectively. A third aliquot was further divided into two aliquots for knockdown of *OR2C1*, among which one aliquot was transfected with 20 nM siRNA OR2C1 and siRNA NC for 12 h and another one was additionally incubated with heparin for 1 h after transfection.

### 4.4. Assessment of Acrosomal Status and Capacitation Rate

Double staining with FITC conjugated with PSA and PI was applied to evaluate acrosomal status and membrane integrity as previously described [67]. In brief, after being fixed in 4% paraformaldehyde for 10 min, sperm were subsequently incubated with FITC-PSA (20 µg/mL) for 20 min and PI (20 µg/mL) for 5 min at 37 °C. Then, the suspension was washed three times with PBS. For each sample, no less than 200 sperm were observed using Epifluorescence microscopy (Olympus, Tokyo, Japan). Image J (1.48v) was used to overlap the red (PI) and green (FITC-PSA) fluorescence.

### 4.5. RNA Extraction and Quantitative Real-Time PCR (RT-qPCR)

Total RNA was extracted with Trizol LS according to previous method and somatic cells was eliminated by treating with 0.5% of Triton-X hypotonic solution [35]. The Nanodrop 2000 (Thermo Fisher Scientific, Waltham, MA, USA) was used to measure RNA concentration and purity. Qualified RNA sample was converted to cDNA using TaKaRa PrimeScript RT Reagent Kit according to the manufacture protocol. RT-qPCR was used to detect the expression of target genes through CFX 96 Real-Time PCR Detection System (Bio-Rad, Hercules, CA, USA). The *GAPDH* were regarded as the housekeeping genes for normalizing to the relative mRNA expressions.

### 4.6. Homology Model Building and Molecular Docking

The three-dimensional structure of the human M2 muscarinic receptor (PDB code 3UON) was taken as the template to simulate the structure of olfactory receptor proteins as previously described [68]. Modeler modeling 10.3 based on the MPI library was used to construct homology modeling for olfactory receptor [69]. The reliability of those olfactory receptor modellings was examined by the PROCHECK v.3.5. After being evaluated and conforming to the stereochemical energy rules, models were applied to the subsequent molecular docking. The structure of heparin was mapped in ChemDraw 14.0. The MOPAC 2016 was used to optimize the molecular structure and calculate the atomic charge of PM3. The PDBQT file for docking was generated via AutoDock Tools 1.5.6. Molecular docking was achieved by the software package AutoDock 4.2.6. The parameters were set as follows: the lattice points (60 × 60 × 60) in each direction of X, Y, and Z, 100 docking times, and the other parameters were adopted to the default values. Amber14 force field was used to optimize docking results. Briefly, the steepest descent method of 1000 steps were taken, followed by the conjugate gradient method of 500 steps, and the final results will be used for subsequent analysis.

### 4.7. Molecular Dynamic Simulations

Molecular dynamics (MD) simulation of OR2C1 and heparin were performed with the GROMACS 2018.4 version package in an isothermal–isobaric ensemble (NPT). The Amber14SB all-atom force field [70] and TIP3P water model [71] were used in this system. All bonds involving hydrogen atoms were constrained by the LINCS algorithm [72] with a 2 fs integration time. Electrostatic interactions were calculated using the PME method (Particle Mesh Ewald) [73], and the cut-off radius of the non-bonded interactions was set as 10 Å, which was updated every 10 steps. The V-Rescale [74] temperature coupling method was used to control the simulated temperature to 298.15 K, and the Parrinello–Rahman [75] method was employed to control the pressure to 1 bar. Firstly, the steepest descent method is used to minimize the energy of the two systems. Then, canonical ensemble (NVT) and NPT equilibria were simulated for 1 ns at 298.15 K, respectively. Finally, the system was simulated by MD for 100 ns, and the conformation was saved every 10 ps. The simulation results were visualized using the GROMACS embedding program and VMD.

### 4.8. Dual-Glo Luciferase Assay

The full-length genomic of *OR2C1* was amplified by the specific primers tag with N-terminal rhodopsin and was subcloned into pCI vector between Mlu I and Not I restriction enzyme sites. The sequence and primers of *OR2C1* gene of pig were shown in Figure S2. Hana3A, derived by HEK239T, was cultured in RPMI medium supplemented with 10% FBS (vol/vol) at 37 °C. The constructed pCI was co-transfected with three plasmids in Hana3A: firefly luciferase driven by CRE, sea kidney luciferase driven by the constitutive promoter SV40, and RTP1S driven by CMV like the olfactory receptor using TransIntro^TM^ PL transfection reagent. RTPIS was used to promote olfactory receptor surface expression. Subsequently, the cells were treated with heparin for 4h. Luciferase activity was measured in Hana3A cells with the Dual-Glo Luciferase Assays System.

### 4.9. Knock-Down of OR2C1

To reduce the expression of *OR2C1* mRNA, the Cell Manipulation ECM-2001 (BTX, Holliston, MA, USA) was used to transiently transfect sperm with siRNA oligonucleotides targeting *OR2C1* mRNA. Pulse conditions were set to 4 × 300 V for 100 µs. The level of *OR2C1* expression and capacitation rates were determined after siRNA knock-down.

### 4.10. Statistical Analysis

All experiments were performed independently in at least triplicate and results were presented as mean ± standard error of mean (SEM). The statistical difference was analyzed by independent sample *t*-test using SPSS (v.19.0). The relative olfactory receptors genes expression level was quantified by the 2^−∆∆CT^ method. *P* < 0.05 and *p* < 0.01 was considered statistically significant.

## 5. Conclusions

At the beginning of present study, we demonstrated that heparin extensively upregulated mRNA expression of ORs. Further, we revealed the potential association between OR2C1 and heparin by knocking down OR2C1 during heparin-induced sperm capacitation. We also confirmed that OR2C1 was involved in the regulation of sperm capacitation by binding with heparin and identified its potential crucial binding site. Overall, we comprehensively provided novel evidence that heparin induces sperm capacitation via binding with OR2C1 located in the plasma membrane, which will contribute to elucidating the molecular regulatory mechanism of sperm before reaching the oocyte.

## Figures and Tables

**Table 1:** Binding free energy between heparin and OR2C1.

Item	Energy (kcal/mol)	Delta
ΔG_vdw_ (kcal/mol)	−35.293	3.934
ΔG_ele_ (kcal/mol)	−318.485	41.848
ΔG_PB_ (kcal/mol)	318.227	37.706
ΔG_np_ (kcal/mol)	−5.193	0.349
ΔG_bind_ (kcal/mol)	−40.744	9.973

ΔG_vdw_: van der Waals force interaction; ΔG_ele_: electrostatic interaction under vacuum; ΔG_PB_: polar solvation energy; ΔG_np_: non-polar solvation free energy; ΔG_bind_: bingding free energy.

## Data Availability

Not applicable.

## Supplementary Materials

The supporting information can be downloaded at: https://www.mdpi.com/article/10.3390/ijms24021664/s1.
