# Supplementary material for: Odorant Receptor OR2C1 Is an Essential Modulator of Boar Sperm Capacitation by Binding with Heparin
Source: Int J Mol Sci. 2023 Jan 14;24(2):1664. doi: 10.3390/ijms24021664 (PMC9861704; doi:10.3390/ijms24021664)
Supplement: Supplementary file 1 [file ijms-24-01664-s001.zip › Supporting Information.pdf]

Supporting Information

OR2C1 is a novel target binds heparin modulating sperm capacitation

Xiang Yuan[1,†], Yihan Wang[1,†], Malik Ahsan Ali[1,2], Ziyue Qin[1], Zhihua Guo[1], Yan Zhang[1], Ming Zhang[1], Guangbin Zhou[1], Jiandong Yang[1], Lei Chen[1], Linyuan Shen[1], Li Zhu[1], Changjun Zeng[1,*]

[1] College of Animal Sciences and Technology and Farm Animal Genetic Resources Exploration and Innovation Key Laboratory of Sichuan Province, Sichuan Agricultural University, Chengdu, Sichuan, 611130, China;

2 Department of Theriogenology, Riphah College of Veterinary Sciences, Lahore, 54000, Punjab, Pakistan;

¶ These authors contributed equally to this work.

***** Correspondence: Changjun Zeng, zengchj@sicau.edu.cn

Keywords: Olfactory receptor; OR2C1; Heparin; Boar sperm; Capacitation

Supplementary Tables

Table S1: The homology model (a), ramachandran plot (b) and docked structure of proteins with heparin.

Protein	Model and Ramachandran plot	Docked Structures
OR2C1	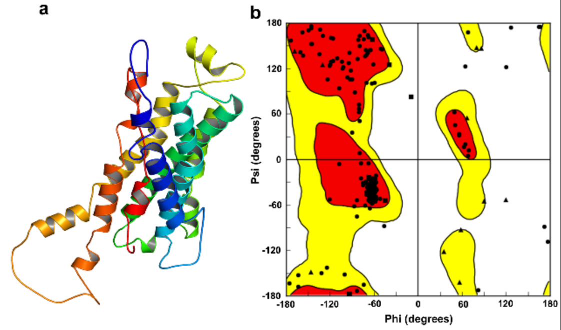	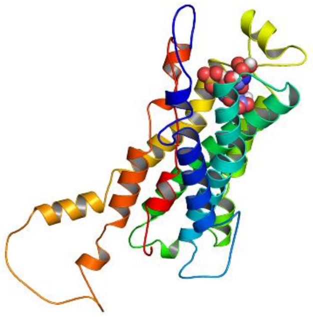
OR1L8	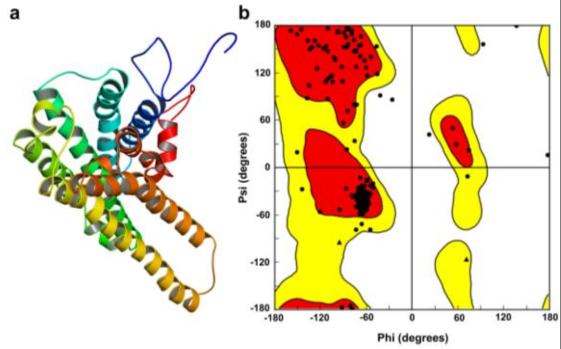	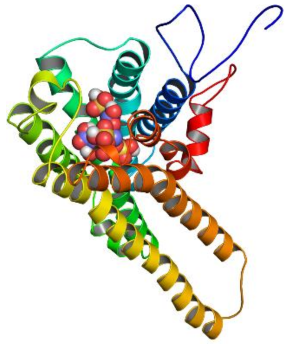
OR2M3	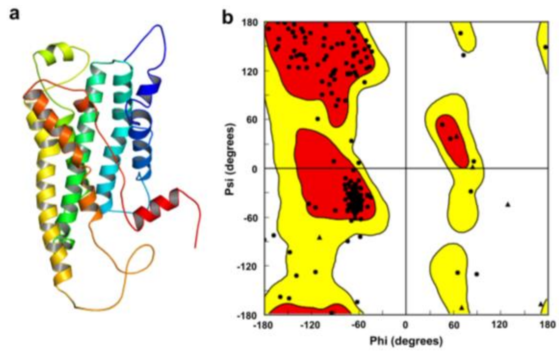	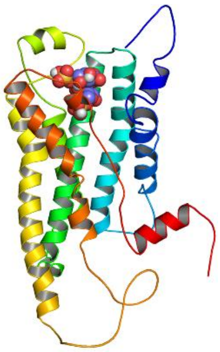
OR4K13	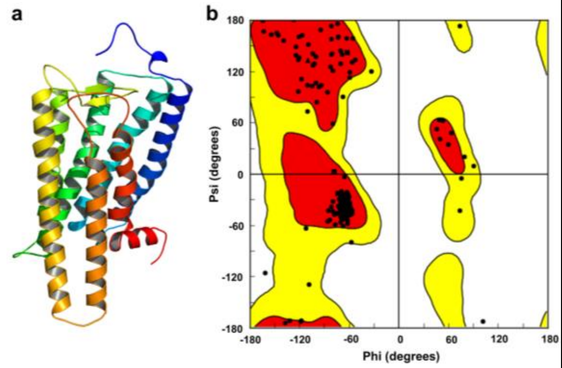	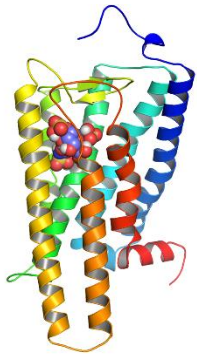

OR5V1	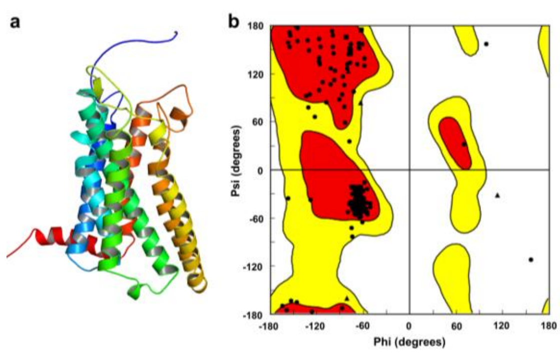	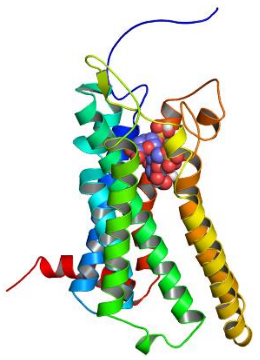
OR6S1	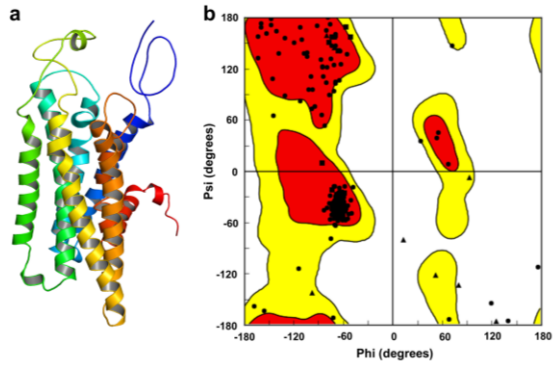	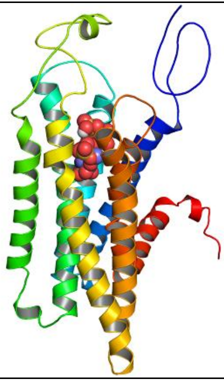
OR7A17	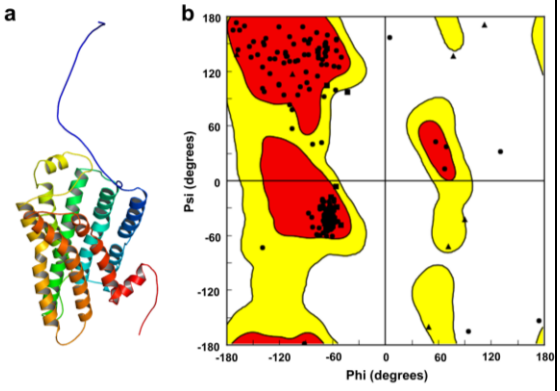	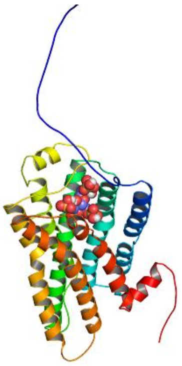
OR10Z1	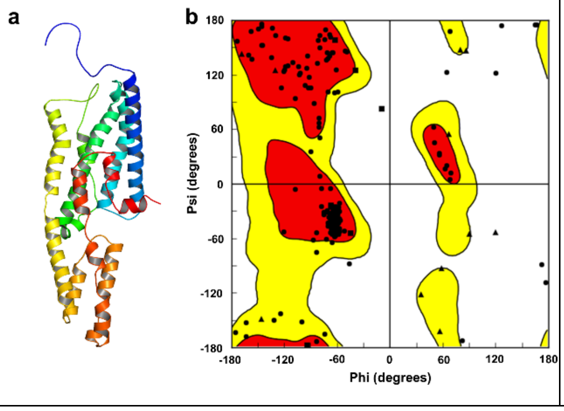	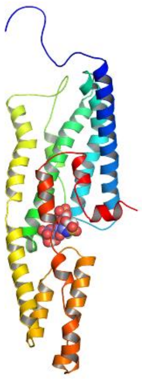

OR49	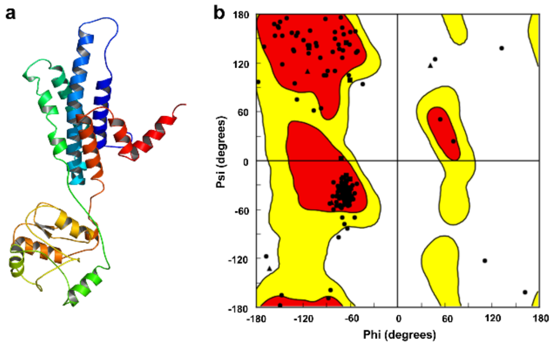	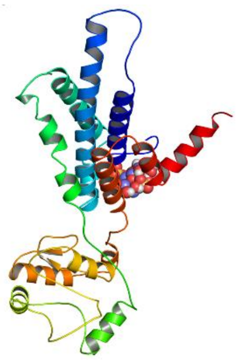
OR51F2	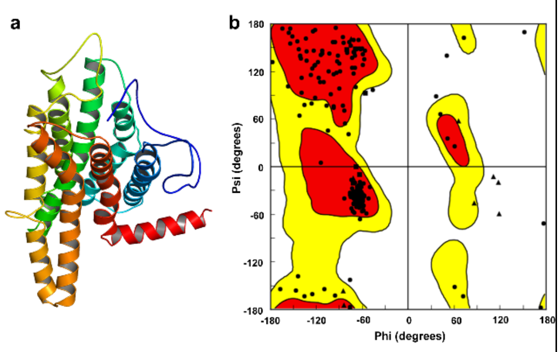	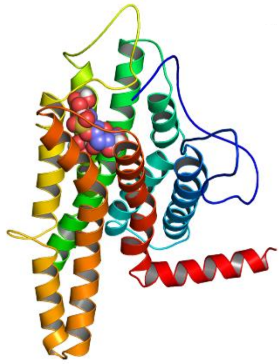
OR51T1	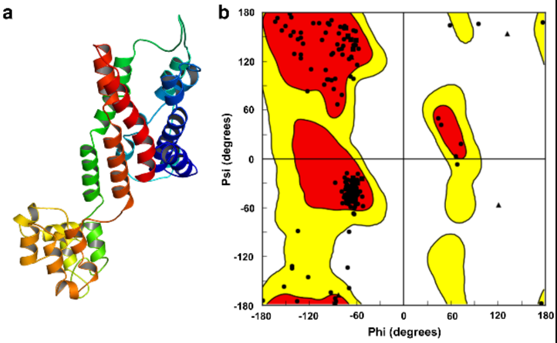	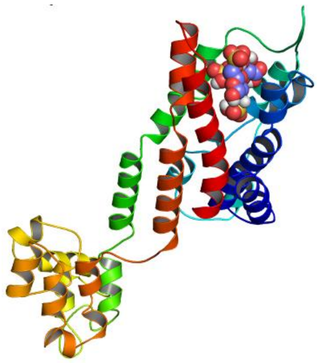
OR52E4	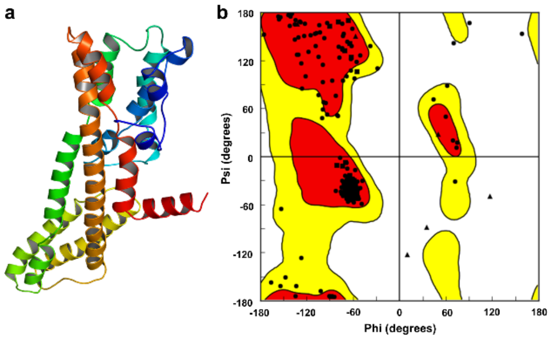	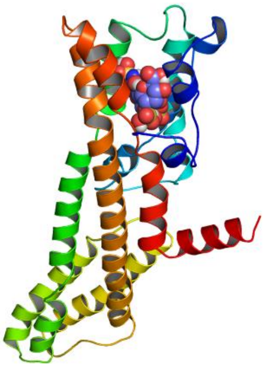

OR52K1	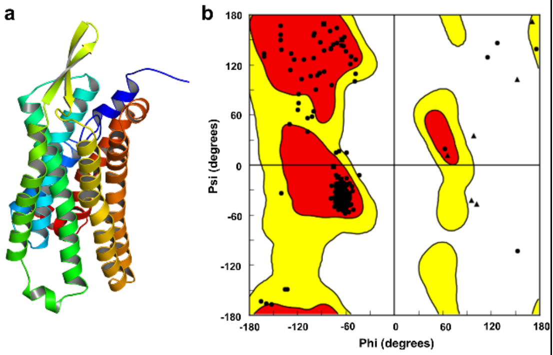	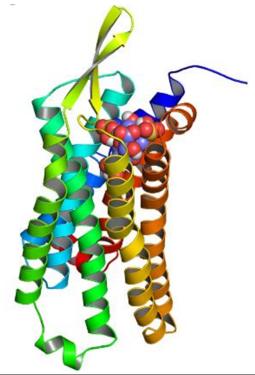
OR140	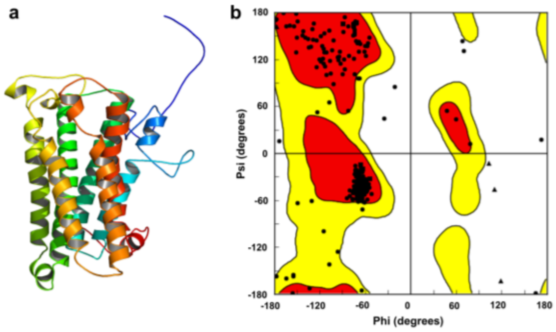	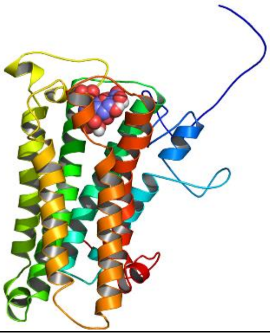

Table S2: Docking score and detailed indicators of olfactory receptor with heparin.

Protein	Docking Score with heparin (kcal/mol)	Intermolecular Energy (kcal/mol)	Electrostatic Energy (kcal/mol)	Internal Energy (kcal/mol)	Predicted *K*i (μM)
OR2C1	-6.79	-12.16	-4.50	-12.12	10.60
OR1L8	-4.98	-10.35	-0.39	-9.46	222.27
OR2M3	-6.25	-11.62	-1.11	-9.29	26.32
OR4K13	-1.63	-7.00	-0.11	-9.99	64.04
OR5V1	-5.85	-7.22	-0.32	-10.27	44.09
OR6S1	-2.33	-7.70	-0.36	-10.41	19.49
OR7A17	-2.88	-8.25	-0.92	-10.66	7.76
OR10Z1	-4.36	-9.73	-2.78	-11.49	632.86
OR49	-5.53	-10.90	-4.36	-6.29	87.75
OR51F2	-4.81	-10.18	-0.70	-10.32	297.59
OR51T1	-6.23	-11.60	-4.84	-10.56	27.04
OR52E4	-4.22	-9.59	-0.79	-11.61	805.57
OR52K1	-5.86	-11.23	-1.46	-10.89	50.35
OR140	-4.39	-9.76	-3.85	-9.94	602.51

Supplementary Figures

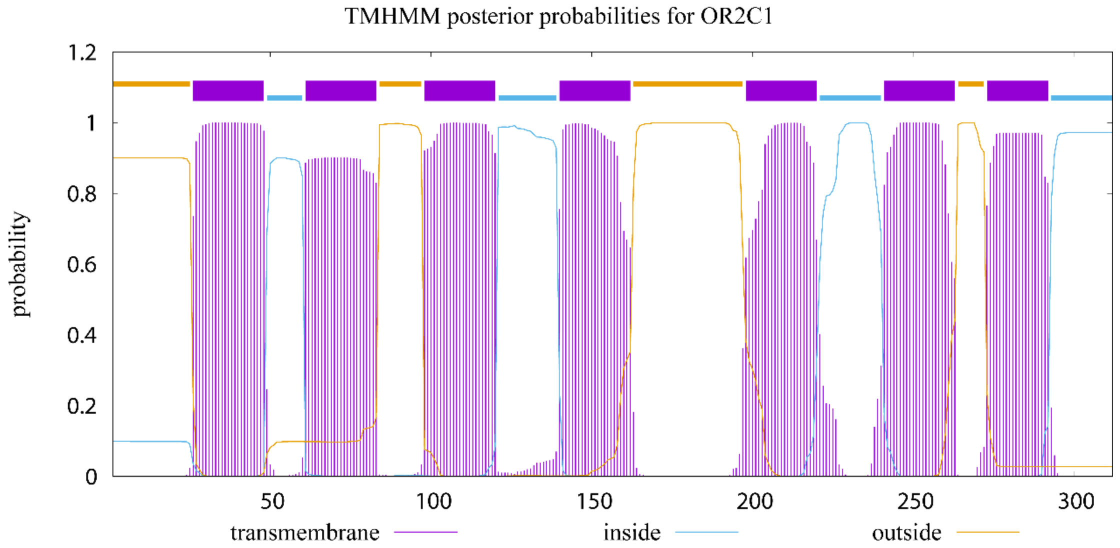

Figure S1: TMHMM posterior probabilities for OR2C1. Seven transmembrane regions are clearly indicated as purple bar.

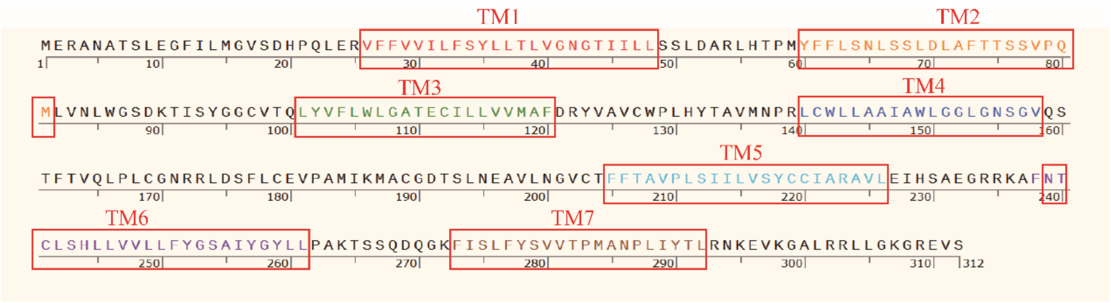

Figure S2: The full residue sequence of OR2C1 protein, the different TM region is marked in the different colors and red rectangle box.

.
